# Rapid inversion of singleton distractor representations underlies learned attentional suppression

**DOI:** 10.1101/2025.10.08.680699

**Published:** 2025-10-09

**Authors:** Ziyao Zhang, Jarrod A Lewis-Peacock

**Affiliations:** Department of Psychology, The University of Texas at Austin

## Abstract

In visually complex and dynamically changing environments, humans often face the challenge of filtering out salient stimuli that are presently irrelevant to their tasks. Recent evidence suggests that through repeated exposure to search arrays containing salient color singleton distractors, individuals can learn to divert their attention away from such salient but irrelevant stimuli, even before they capture attention. However, the mechanisms underlying such attentional suppression remain unclear. The current study examined trajectories of singleton distractor representations during visual searches to address this gap. Using multivariate pattern analyses on EEG data (N = 40), we found that singleton distractor representations underwent a rapid inversion approximately 200 ms into the search. These inverted representations were coded in a shared subspace with target representations, but in a reversed orientation, presumably to downweight their salience in the spatial priority map. Target locations were consistently enhanced compared to non-singleton distractors, while singleton distractors were suppressed. Our findings reveal a novel mechanism of rapid representational transformation underlying salient distractor suppression at the onset of visual search. The rapid inversion of pop-out singleton distractor signals resulted in an inverted arrangement of target and distractor representations in a shared neural subspace, which facilitates subsequent read-out of both target enhancement and distractor suppression signals in the computation of spatial priorities to successfully guide search.

## Introduction

The ability to filter out distracting information is crucial for goal-directed behavior, particularly in today’s digitally saturated environment. Stimuli such as website pop-ups inherently capture attention, raising the question of whether salient stimuli can be effectively suppressed within the visual system to support goal-directed actions.

Recent insights into this debate stem from research on suppressing color singleton distractors (e.g., a red item among green items) in goal-oriented search tasks (Bacon & Egeth, 1994; Gaspelin & Luck, 2018b, 2018a; Luck et al., 2021). Color singleton distractors readily capture attention and impede visual searches (Jonides & Yantis, 1988; Theeuwes, 2010). However, emerging evidence suggests that proactive inhibitory processes can mitigate attentional capture by color singleton distractors. Repetitive training on search arrays containing color singleton distractors led participants to be faster at detecting a shape target when a color singleton distractor was present, compared to when it was absent (Chang & Egeth, 2019; Gaspelin et al., 2015, 2017; Vatterott & Vecera, 2012). Eye-tracking data indicated that initial saccades were less likely to land on singleton distractors than on non-singleton distractors (Gaspelin et al., 2017; Stilwell et al., 2023). Additionally, an ERP component, Pd was identified in tasks with observed behavioral attentional suppression, suggesting that the suppression of color singleton distractors might be associated with early inhibitory processes occurring around 100–300 ms into the search (Cosman et al., 2018; Gaspelin et al., 2023; Kerzel & Burra, 2020; Sawaki et al., 2012; Sawaki & Luck, 2010). These findings have led to the proposal of the *signal suppression hypothesis* (Gaspelin & Luck, 2018b; Sawaki et al., 2012; Sawaki & Luck, 2010), which posits that physically salient stimuli automatically generate a bottom-up signal that can capture attention, but top-down mechanisms can suppress them to prevent attentional capture.

Despite significant progress in the attentional capture debate, the neural mechanisms underlying attentional suppression remain unclear. Neural recordings and imaging studies have suggested two potential mechanisms for attention suppression. First, suppression is likely supported by attenuation of distractor-related signals. Singleton distractor signals may be inhibited during the initial stage of sensory processing due to adaptation (Turatto et al., 2018; Vatterott & Vecera, 2012; Won et al., 2019). Consistent with the adaptation account, an fMRI study demonstrated that repeated singleton distractors were suppressed starting in V1. In contrast, goal-driven target enhancement effects were absent in V1 but became more prominent in the intraparietal sulcus (IPS, Adam & Serences, 2021). These findings suggest that target enhancement is mediated by feedback signals originating in prefrontal control regions and projecting to parietal areas, whereas singleton distractor suppression may be driven by reduced feedforward activity beginning in V1. Neural recordings in non-human primates (NHPs) have shown that singleton distractor signals are inhibited in regions responsible for computing spatial priorities (Cosman et al., 2018; Ipata et al., 2006). Recordings of neuronal activities in the lateral intraparietal area (LIP) and frontal eye fields (FEF) in NHPs have shown that color singleton distractors elicited weaker activities when appearing in the receptive field of neurons compared to non-singleton distractors. This suggests that suppression of singleton distractors occurs in regions implicated in spatial priority computations.

In contrast to adaptation-like signal attenuation, attentional suppression might also be supported by signal transformation mechanisms. A recent study showed that salient singleton distractors showed enhanced neural activity early during the search, followed by later suppression compared to non-singleton distractors in the V4 region of NHPs around 150 ms into the search (Klink et al., 2023). This finding of initial enhancement but later suppression of singleton distractors aligns with the original description of the signal-suppression hypothesis, which suggests that attentional suppression involves inhibiting the initial priority signals generated by color singleton distractors (Gaspelin & Luck, 2018b; Sawaki & Luck, 2010). This transformation from an initial priority signal to an eventual suppression signal has long been implied and widely accepted, yet direct evidence has been limited (Klink et al., 2023). Here, we provide neural evidence supporting this transformation.

The mechanisms underlying the potential signal transformation of distractors remain both puzzling and intriguing. To examine this, we used temporal generalization analyses of EEG data to test potential representational shifts during a distractor-filled visual search process. Temporal generalization is particularly useful for disentangling signal attenuation from signal transformation as potential mechanisms of attentional suppression. The signal attenuation account suggests that distractor signals gradually weaken during the search, but should maintain neural representations that can generalize across time. Conversely, the signal transformation mechanism posits that singleton distractor representations dynamically change during the search, leading to orthogonal or even negatively correlated neural representations across time. To test these competing hypotheses, we utilized multivariate pattern analysis (MVPA) on EEG data to assess changes in singleton distractor representations during a visual search task.

## Results

Participants searched for a predefined target item (circle or diamond, counterbalanced between participants) in a search array. In the search array, the inner ring contained a target shape and distractors that were in different shapes. On 75% of trials, a color singleton distractor that had a distinct color from other items was presented in the inner ring. The color singleton distractor was never the target, and participants were instructed to use the shape feature to locate the target item. The outer ring contained non-target shapes only to boost the relative salience of the singleton distractor.

### Singleton distractor representations showed distinct temporal patterns compared to target representations.

**Fig. 1.**
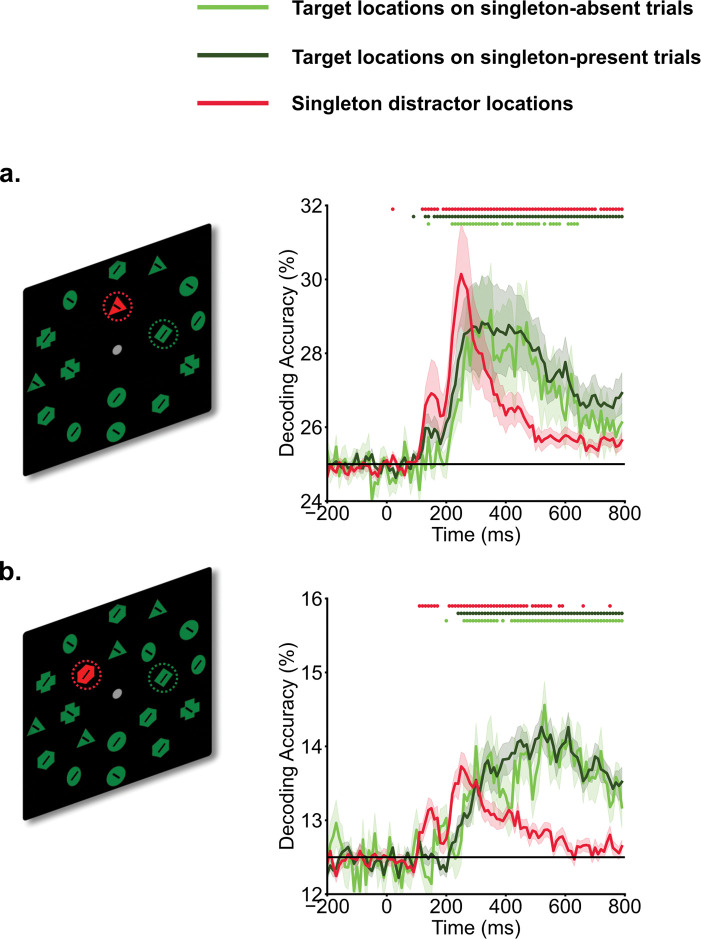
Temporal profiles of target and singleton distractor representations. Participants searched for a specific target shape (e.g., a green diamond) within the inner ring of a search array, and made a speeded button press to indicate the direction of the line within the target shape (left vs. right). Time 0 ms represents the onset of search arrays. In experiment 1 (a. set size 4) and experiment 2 (b. set size 8), decoding of singleton distractors showed an earlier initial peak of decoding evidence from 100 ms to 200 ms, followed by a later peak from 200 ms to 400 ms. Target decoding showed a gradual increase in evidence The horizontal black line indicates chance-level (25% for set size 4, and 12.5% for set size 8). Shaded areas indicate standard errors. Colored dots indicate above chance-level decoding performance (*p* < .05, one-sided t test).

We applied MVPA to track target and singleton distractor representations across time. Both singleton distractor and target locations showed successful decoding (Fig 1). Target location representations emerged around 200 ms and persisted for most of the search period. Interestingly, the decoding of singleton distractor locations revealed an earlier initial peak, spanning from 100 ms to 200 ms. This initial peak was followed by a subsequent, more robust peak occurring between 200 ms and 400 ms for both set size 4, and set size 8. The decoding accuracies of singleton distractor locations quickly dropped off following the second peak whereas that for target locations persisted.

**Fig. 2.**
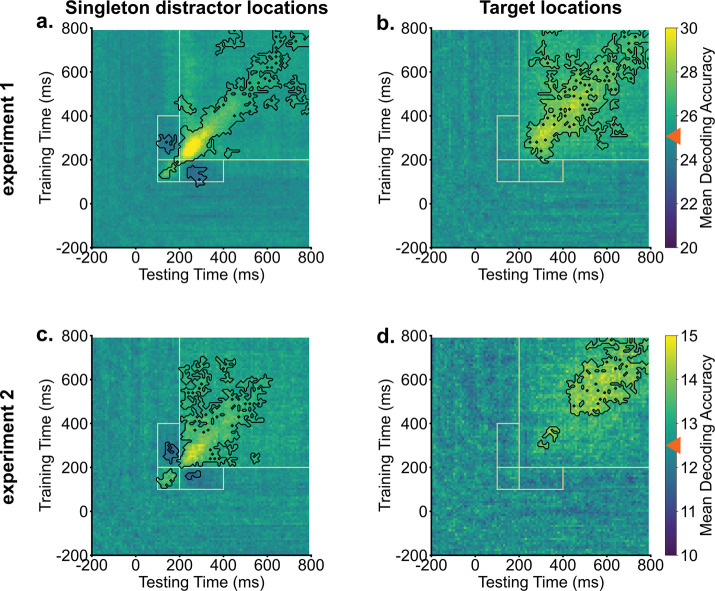
Singleton distractor representations showed inverted transformations, whereas target representations remained stable. Across experiments, generalization analyses of singleton distractor representations (a & c) revealed two clusters. Training decoders on activities from 100 ms to 200 ms resulted in below chance-level decoding evidence when tested on activities from 200 ms to 400 ms, and vice versa. Target representations demonstrated stability and could be generalized to neighboring time points (b & d). Orange boxes indicate key generalization time windows. Black contours indicate significant areas (*p* < .05). Bright colors indicate above chance-level decoding performance (denoted by orange triangles), and dark colors indicate below chance-level decoding performance.

The identification of two early peaks in singleton distractor decodings suggests potential shifts in representation during the search process. We conducted temporal generalization analyses to further examine these potential changes. Our methodology involved training decoders at one time point and then testing the decoder across all time points, repeating this procedure for each time point. As shown in Fig 2, target representations were stable over time. Trained decoders were able to generalize to neighboring time points. In contrast, the temporal generalization analysis of singleton distractor representations showed two discernible clusters. The initial cluster extended approximately from 100 ms to 200 ms, while the subsequent cluster emerged at around 200 ms. Crucially, these two clusters showed an inverted relationship. Decoders trained on activity from 100 ms to 200 ms had below-chance decoding performance when tested on data from 200 ms to 400 ms and vice versa. This negative generalizability performance suggests an inversion of representations for singleton distractors between the early (100 – 200 ms) and the later (200 – 400 ms) time windows. It also provides preliminary evidence for the signal transformation hypothesis that neural codings of singleton distractors were inverted during the search. However, it is unclear yet how this inversion directly supported attentional suppression.

### Singleton distractor locations were suppressed in the spatial priority map.

**Fig. 3.**
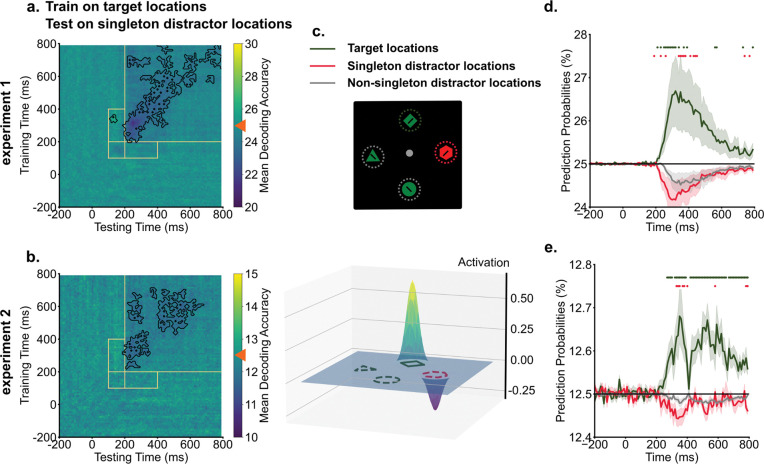
Singleton distractor locations were suppressed in the spatial priority map. a & b) Across experiments, training decoders on target locations from singleton-absent trials demonstrated below chance-level decoding performance (denoted by orange triangles) for singleton distractor locations, indicating the suppression of singleton distractor locations. Dark colors indicate below chance level decoding performance for singleton locations. Black contours indicate significant areas (*p* < .05). c) An example (250 ms in experiment 1) illustrates how we computed activation scores from decoding prediction probabilities. Prediction probabilities of target locations (green) and singleton distractor locations (red) were baselined to the averaged prediction probabilities of non-singleton distractor locations (gray). d & e) The time course of prediction probabilities for target locations and singleton distractor locations were plotted against the baseline (other locations). From 200 ms into the search, target locations showed increased prediction probabilities, whereas singleton distractor locations showed decreased prediction probabilities compared to the baseline. Shaded areas indicate standard errors. Colored dots indicate significant differences in prediction probabilities between target, singleton distractor locations to non-singleton distractor locations (*p* < .05).

To investigate how the inversion of singleton representations supported attentional suppression and facilitated visual search, we performed cross-condition generalization analyses. In this procedure, decoders were trained on target representations in singleton-absent trials, where top-down enhancements served as the primary guiding sources to boost target locations in the priority map. Subsequently, these trained decoders were tested on singleton-present trials to reveal the priority maps in instances where both target enhancement and singleton suppression might influence spatial priorities. The cross-condition generalization analyses revealed that singleton distractor locations were suppressed in the spatial priority map, resulting in below chance-level decoding performance (Fig 3.a & b). This suppression of singleton distractor locations started around 200 ms and persisted. Intriguingly, no generalization was observed between target representations and singleton distractor locations during the early time window from 100 ms to 200 ms, corresponding to the initial cluster of singleton distractor representation observed in temporal generalization analyses.

Below-chance decoding performance could reflect suppression of singleton distractor locations. Alternatively, it might be driven by a strong enhancement of target locations, which would reduce decoding performance for all other locations. To rule out the alternative possibility, we compared the spatial priority of singleton distractors against a baseline of non-singleton distractors in the search array. We derived prediction probabilities from trained decoders for each location in the search array and compared them across target locations, singleton distractor locations, and non-singleton distractor locations. Activation scores for target and singleton distractor locations were computed as the difference in prediction probabilities compared to the baseline (non-singleton distractor locations, for one example time point, see Fig 3.c). For between 200 ms and 400 ms into the search, we found enhancement of target locations compared to the baseline [experiment 1: *t*(19) = 2.14, *p* = 0.045, *d* = 0.81, *BF10* = 1.51; experiment 2: *t*(19) = 2.93, *p* = 0.009, *d* = 1.02, *BF10* = 5.78]. Critically, suppression of singleton distractor locations compared to the baseline was also observed across the experiment [experiment 1: *t*(19) = −2.11, *p* = 0.048, *d* = 0.32, *BF10* = 1.42; experiment 2: *t*(19) = −2.62, *p* = 0.017, *d* = 0.68, *BF10* = 3.30]. As shown in Fig 3.d & e, prediction probabilities of target locations displayed enhancement relative to the baseline from 200 ms onwards. In contrast, prediction probabilities of singleton distractor locations were suppressed below the baseline.

### Singleton distractor and target locations were coded in a shared subspace but in a reversed manner.

Cross-condition generalization analyses suggested that singleton distractors were suppressed in the neural subspace representing target locations. We hypothesized that this suppression might be driven by coding distractor information in an inverted format relative to target information. Such inverted coding of target and distractor information could facilitate the integration of top-down enhancement and suppression in computing spatial priority (Awh et al., 2012; Fecteau & Munoz, 2006; Itti & Koch, 2000; Luck et al., 2021). To directly test this hypothesis, we conducted neural correlation analyses to examine the relationship between target and singleton distractor representations.

**Fig. 4.**
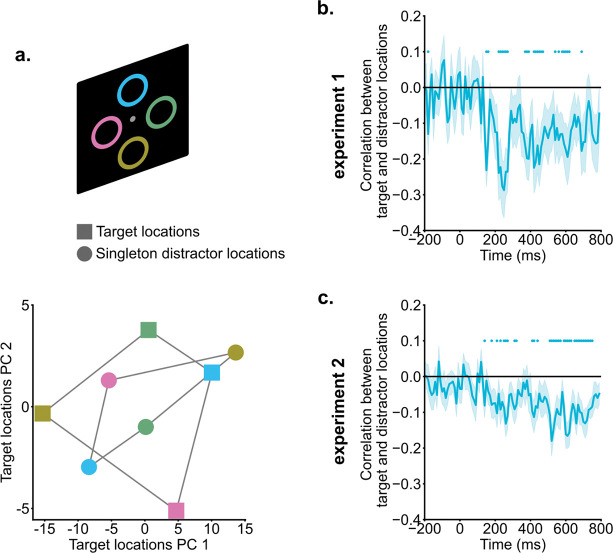
Singleton distractor suppression and target enhancement relied on inverted neural coding. a) Singleton distractor and target representations of an example subject were projected onto a 2D target representational space for illustration purposes. Different colors indicate different locations. Singleton distractor and target representations exhibited an inverted pattern. b & c) Correlations between singleton distractor and target representations. Starting from around 200 ms, singleton distractor and target representations showed negative correlations. Shaded areas indicate standard errors. Colored dots indicate significant correlations compared to zero (*p* < .05).

In Fig 4.a, representations of both targets and singletons were shown for an example subject from 200 ms to 400 ms into the search, projected in a 2D subspace for easier visualization. This subspace was constructed using the first two principal components (a total of 99% variance explained) of the neural representations of target locations. The location information demonstrated clear organization; the responses to four locations were well separated, and the distance between neural representations corresponded closely to the physical distance between locations (i.e., neighboring locations had neighboring representations). Interestingly, singleton distractor and target representations showed a reversed pattern. For instance, the top location (blue square) in the target representational space was projected to the top-right corner, whereas the same location in the singleton distractor representational space was projected to the bottom-left region (blue circle).

Formal correlation analyses were performed to link target representations and singleton distractor representations in the raw signal space (dimension = channel). For between 200 ms and 400 ms into the search, target and singleton distractor representations showed reliable negative correlations [experiment 1: *t*(19) = −3.22, *p* = 0.005, *d* = 0.72, *BF10* = 9.99; experiment 2: *t*(19) = −3.05, *p* = 0.006, *d* = 0.68, *BF10* = 7.29]. As shown in Fig 4.b &c, negative correlation emerged around 200 ms and persisted into the later search period, suggesting that singleton distractor representations and target representations were coded with an inverted representational geometry.

## Discussion

In recent years, attention research has shifted from a primary focus on target enhancement to an increasing interest in distractor suppression (Geng et al., 2019; Luck et al., 2021; Theeuwes, 2025; Wöstmann et al., 2022). While growing evidence shows that attention can be directed away from cued or learned distractors (Arita et al., 2012; Chang & Egeth, 2019; Gaspelin et al., 2015, 2017; Sawaki & Luck, 2010; Vatterott & Vecera, 2012; Won et al., 2019; Zhang et al., 2020, 2022; Zhang & Carlisle, 2023), it remains unclear how such suppression unfolds over time, particularly how to-be-suppressed information is represented in the brain. The primary goal of this study was to test a novel mechanism of signal transformation underlying singleton distractor suppression in visual search. Using MVPA applied to EEG data, we compared the temporal trajectories of target and singleton distractor representations. We found that singleton distractor representations were reliably represented earlier than targets, consistent with the idea that salient distractors elicit rapid bottom-up signals before top-down target enhancement emerges (Jonides & Yantis, 1988; Theeuwes, 2010). Increasing set size from four to eight items delayed target representations but did not affect singleton distractor representations, in line with the finding that crowding weakens target signals (Reddy & VanRullen, 2007), whereas salience-based singleton signals remain stable or even enhanced.

We identified two distinct peaks in decoding evidence for singleton distractor locations. To examine potential changes in representations associated with the two peaks, we conducted temporal generalization analyses. Target representations remained stable across time, forming a consistent cluster from around 200 ms to subsequent times in the search. In contrast, singleton distractor representations showed two separate clusters: an early one from 100 ms to 200 ms, followed by a larger cluster from 200 ms to later search periods. Importantly, these two clusters of singleton distractor representations showed negative generalizability, indicating an inverted transformation of coding formats around 200 ms in the search.

We next examined how the inversions of singleton distractor representations impact spatial priority computations. Decoding models were trained on target locations in singleton-absent trials to capture spatial priorities predominantly guided by top-down target enhancement. Then, these trained decoders were applied to data from singleton-present trials to reveal spatial priorities in those trials. This cross-condition generalization analysis showed below-chance level decoding performance for singleton distractor locations. Below-chance decoding performance could reflect an enhancement of target representations, which would drive the prediction probabilities of all non-target locations below-chance. Alternatively, it could result from the suppression of the specific singleton distractor location. To test those possibilities, we calculated prediction probabilities for each location in the search array and used the averaged prediction probabilities of non-singleton distractor locations (two in experiment 1 and six in experiment 2) as the baseline. Compared to this baseline, target locations showed above-baseline enhancement from around 200 ms onwards. Crucially, singleton distractor locations displayed below-baseline suppression around the same time, providing robust evidence for the idea that singleton distractor representations were suppressed in the same neural subspace coding target information.

Finally, we turned to the pivotal question of how singleton distractor representations were suppressed. We hypothesized that suppression is driven by coding singleton distractor representations in an inverted format relative to target representations. Such inverted coding could facilitate the downstream readout and integration of both target enhancement and distractor suppression. To test this idea, we conducted correlations between singleton distractor and target representations. The analysis revealed robust negative correlations starting around 200 ms into the search, indicating that target locations and singleton distractor locations were indeed inversely coded within a shared subspace. This shared subspace likely plays a critical role in the computation of spatial priorities, where target signals and singleton distractor signals generate opposing enhancement and suppression effects. While these findings offer valuable insights, further evidence is required to rigorously test the relationship between the target and singleton distractor subspaces and the hypothesized shared spatial priority map.

**Fig. 5:**
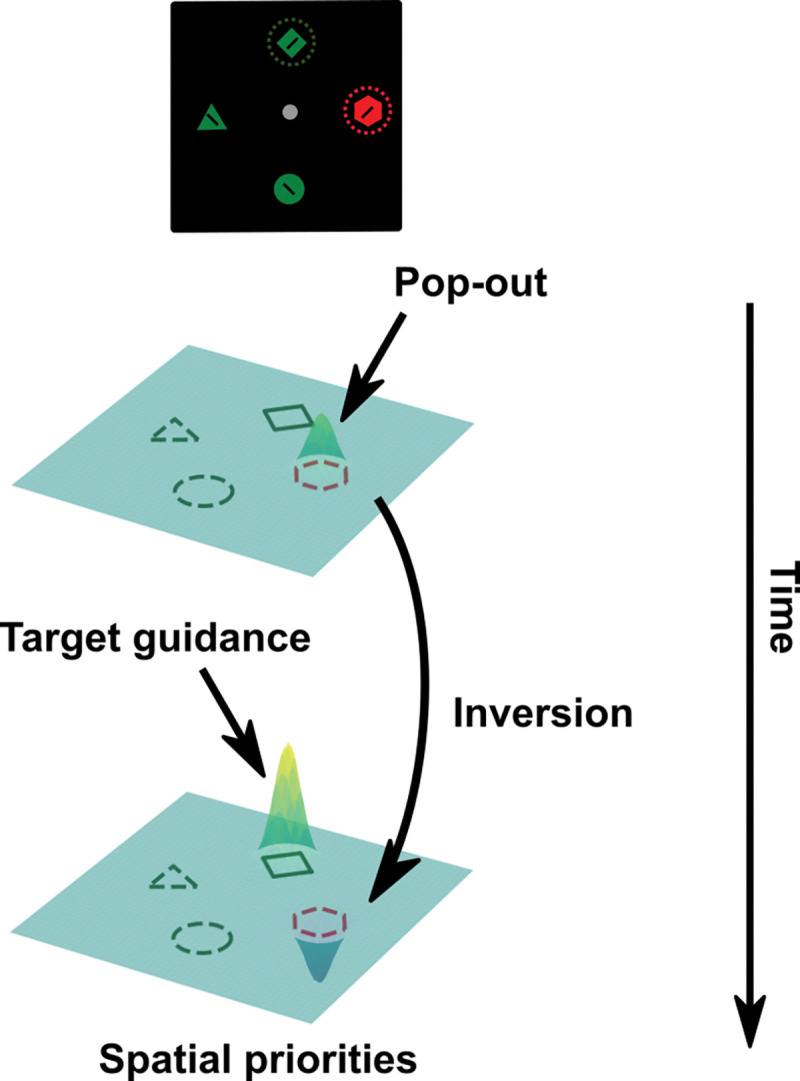
Proposed singleton suppression mechanism within the priority map framework. The process initiates with the registration of singleton distractor information. Subsequently, distractor representations undergo inversion, around the same time as the emergence of target representations. Both distractor and target representations are coded within a shared subspace but in an inverted manner. The representations within this subspace are then accessed to compute the ultimate spatial priorities that guide visual attention.

While accumulated evidence from various modalities, including behavioral, eye-tracking, electrophysiological, EEG, and fMRI data supports the successful suppression of salient color singleton distractors, the neural mechanisms underlying this phenomenon remain less clear (Chang & Egeth, 2019; Cosman et al., 2018; Gaspelin et al., 2015, 2017; Sawaki et al., 2012; Sawaki & Luck, 2010; Stilwell et al., 2022; Vatterott & Vecera, 2012). Previous electrophysiological studies in NHPs have demonstrated that neuronal activity elicited by salient singleton distractors can be suppressed relative to the activity elicited by non-singleton distractors in areas of FEF, LIP, and V4 (Cosman et al., 2018; Ipata et al., 2006; Klink et al., 2023). Additionally, human fMRI studies have indicated that representations of singleton distractors are suppressed across multiple early visual regions and parietal regions involved in computing spatial priorities (Adam & Serences, 2021). This present study extends beyond these prior findings by examining potential representational transformations in singleton distractor processing. In line with previous research, we found evidence of singleton distractor suppression in the computation of spatial priority. Notably, our study revealed a rapid representational inversion of singleton distractor locations early during the visual search that subsequently showed a negative correlation with target representations. These results provide compelling evidence for a representational rotation or inversion mechanism underlying singleton suppression. Based on this evidence, we propose that the attentional suppression mechanism likely involves a rapid transformation of singleton distractor representations into a format subsequently read out as suppression signals in the computation of spatial priority maps (Fig 5).

Recent research by Klink et al. (2023) has reported inversions in neural responses to color singletons, as observed in multiunit activities recorded from V4 in NHPs. Color singletons induced a transient enhancement of V4 activity within the first 100 ms of the search, which was succeeded by sustained suppression. It is noteworthy that in their study, the color of singleton items varied unpredictably from trial to trial, unlike in the current study where singleton colors are fixed and predictable. Previous research (Gaspelin et al., 2017; Stilwell et al., 2023) has shown that when singleton colors are predictable, even the fastest saccades are directed away from the singletons, reflecting the use of a proactive distractor filter rather than a reactive one. Unfortunately, the current dataset lacks eye-tracking data, making it unclear whether the initial cluster of singleton representations signifies attentional capture or some other form of rapid, early processing of the distractor. The question of whether a shared mechanism is involved in the suppression of both predictable and unpredictable singletons remains unanswered (Geng et al., 2019; Won et al., 2019, 2020). However, multivariate analyses of human EEG data and multi-unit recordings in NHPs, using various designs of singleton distractors, have observed a similar pattern of results. This implies that a rapid reversal of singleton distractor representations may function as a common suppression mechanism.

Major hypotheses regarding singleton suppression, including the signal suppression hypothesis (Gaspelin & Luck, 2018b; Sawaki & Luck, 2010) and the rapid disengagement hypothesis (Theeuwes, 2010), both suggest a potential shift from attentional enhancement to suppression signals for singletons, but differ in the hypothesized timing of these transitions. The signal suppression hypothesis proposes that inhibition of the salient singleton happens prior to the initial shift of visual attention (proactive suppression), whereas the rapid disengagement hypothesis suggests that suppression occurs only after singletons have captured attention (reactive suppression). Our temporal generalization analysis identified signal inversion around 200 ms after the onset of the search array which persisted throughout the remainder of the trial. Therefore our results are more consistent with the proactive signal suppression hypothesis, as the onset of top-down guided attention typically occurs around or after 200 ms, regardless of whether the target item is a salient color singleton (Gaspar & McDonald, 2014; Gaspelin et al., 2017; Liu et al., 2022; Woodman & Luck, 1999). Nevertheless, completely ruling out the rapid disengagement hypothesis is challenging, as it remains possible that singleton distractors could lead to very rapid attentional capture that is then quickly suppressed.

It is crucial to note that the initial singleton distractor representations observed from 100 ms to 200 ms are coded in a different format than target representations at any subsequent time point during the visual search. Training decoders on target representations in singleton-absent trials failed to decode those early distractor representations. According to the signal suppression hypothesis, this evidence might indicate that initial singleton representations are associated with registering singleton distractor information in preattentive maps, which does not impact attention unless subsequently incorporated into the priority map (Gaspelin & Luck, 2018b). Conversely, according to the rapid disengagement account, these initial representations of singletons could serve as guiding signals that capture attention (Theeuwes, 2010), although the format of this initial guiding signal differs from that of guiding signals by target representations. Future research is required to elucidate whether guiding signals of top-down goals and bottom-up salience are orthogonal or generalizable.

The efficiency of visual search relies on various guiding signals, including information about relevant goals (goal-driven), previous search experience (experience-driven), and the salience determined by local image statistics (stimulus-driven) (Awh et al., 2012; Kim & Anderson, 2019; Luck et al., 2021; Narhi-Martinez et al., 2023). These diverse guiding signals are integrated somehow into topographically organized priority maps, which direct visual attention based on the relative strength of priority signals among candidate locations (Awh et al., 2012; Fecteau & Munoz, 2006; Itti & Koch, 2000; Luck et al., 2021). While this framework of priority maps is valuable for studying multiple sources of guiding signals, only a limited number of neuroimaging studies have explored how spatial priorities are computed and utilized for guiding attentional suppression (Adam & Serences, 2021; Serences & Yantis, 2007; Sprague et al., 2018; Sprague & Serences, 2013). Our study estimated changes in spatial priorities using cross-condition generalization analyses and we found evidence of singleton suppression. Additionally, we identified a representational subspace (Buschman & Miller, 2007; El-Gaby et al., 2024; Panichello & Buschman, 2021; Xie et al., 2022) in which target and singleton representations were inversely coded. This representational subspace could potentially serve as the neural implementation of the spatial priority map, or the representations within it could be subsequently read out to compute the actual spatial priority map. Distinguishing the final spatial priority map from different subspaces that encode guiding signals from various sources poses a challenge. Future research is needed to investigate how different guiding signals are coded and transformed to compute spatial priorities.

In conclusion, our findings elucidate a novel mechanism of representation inversion underlying singleton distractor suppression in visual searches. We observed a rapid inversion of distractor representations early in the search process. These inverted representations are coded in a shared subspace with target representations but exhibit an inverse relationship, leading to the suppression of singleton locations in the estimates of spatial priorities to guide behavior.

## Materials and Methods

We reanalyzed data shared from Stilwell et al., 2022. Two groups of 20 participants were recruited for experiment 1 and experiment 2, respectively. In each trial, participants searched for a predefined target item (circle or diamond, counterbalanced between participants) in a search array. In the search array, the inner ring contained a target shape and distractors that were in different shapes. On 75% of trials, a color singleton distractor that had a distinct color from other items was presented in the inner ring. The color singleton distractor was never the target, and participants were instructed to use the shape feature to locate the target item. The specific color of the color singleton distractor (red or green) was fixed for each participant but counterbalanced across participants. The outer ring contained non-target shapes only to boost the relative salience of the singleton distractor. Overall, participants completed 1296 singleton-present trials and 432 singleton-absent trials. Search arrays in experiment 1 had an inner ring with four items (effective set size 4), while in experiment 2, the inner ring had eight items (effective set size 8). EEG signals were collected and preprocessed following the procedures outlined in Stilwell et al., 2022.

### Decoding of target and singleton distractor locations

Location decoding was conducted separately for singleton and singleton-absent trials. For singleton-absent trials, we used support vector machine (SVM) to classify the target item’s location based on the spatial distribution of the EEG signal across 17 posterior electrodes (Pz, P3, P5, P7, P9, PO7, PO3, O1, POz, Oz, P4, P6, P8, P10, PO4, PO8, O2). We implemented this model using the *svc* function from the *sklearn* package in Python. EEG data were downsampled to 100 Hz, and decoding was performed for each time point (10 ms) ranging from −200 ms to 800 ms relative to the onset of the search array. For each time point, the dataset was divided into 3 folds. An equal number of trials from each location class were randomly selected for each fold without replacement to ensure unbiased training. Two folds were used as the training set, and the remaining fold served as the testing set. This procedure was repeated for the 3 folds, with each one serving as the testing set in an iteration. The entire process was repeated 100 times (Wolff et al., 2020), and decoding accuracies were averaged across 100 iterations. Decoding of target and singleton distractor locations in singleton-present trials followed the same procedure as decoding target locations in singleton-absent trials, except for adjusting the training set to match the number of trials in singleton-absent trials. This adjustment aimed to ensure equal training data for decoders in both singleton and singleton-absent trials. This should prevent biases in decoding performance comparisons between singleton and singleton-absent trials resulting from uneven training sets.

### Temporal generalization analysis

Temporal generalization analyses were conducted for target locations in singleton-absent trials and singleton locations in singleton-present trials. The same decoding procedures were used, with the only difference being that trained decoders for each time point were used to predict location labels for data at all time points.

### Cross-condition generalization

Cross-condition generalization was performed between target locations in singleton-absent trials and singleton distractor locations in singleton-present trials. SVM decoders were trained exclusively on data from singleton-absent trials with labels indicating target locations. These trained decoders were then used to predict singleton distractor locations in singleton-present trials. Decoding accuracies were computed based on the probability of the decoders’ predictions matching the actual singleton distractor location labels.

### Activation scores

To compute activation scores for each location in singleton-present trials, decoders were first trained on target locations in singleton-absent trials. These trained decoders were subsequently used to predict location labels in singleton-present trials. Instead of generating prediction labels, prediction probabilities were produced to indicate the likelihood of a given location being the true label based on the neural patterns. Prediction probabilities for target locations, singleton locations, and non-singleton distractor locations were averaged across trials for each subject. For plotting the spatial priority map, the averaged prediction probabilities of non-singleton distractor locations (two in experiment 1, six in experiment 2) were used as the baseline activation. The activation for target and singleton distractor locations was computed as the difference between the prediction probabilities of these locations and the baseline.

### Correlations between target and singleton representations

Raw EEG data of singleton-absent trials (200 – 400 ms) with the same target location were averaged to create the mean target representations for each potential target location (four in experiment 1 and eight in experiment 2). Likewise, singleton-present trials with the same singleton distractor location were averaged (200 – 400 ms) to generate singleton distractor representations for all potential singleton distractor locations (four in experiment 1 and eight in experiment 2). Pearson correlations in multi-channel EEG activities between target and singleton representations were computed for each potential location and then averaged. Positive correlations indicate similarity between the location representations in the target space and location representations in the singleton distractor space, while negative correlations suggest inverted representations.

Principal Component Analysis (PCA) was utilized to visualize the relation between the target representational space and the singleton distractor representational space. For this purpose, we identified the first two principal components (PCs) of the target representational space for one example participant (99% variance explained). Both target and singleton distractor representations were projected into the space defined by these two PCs. It should be noted that PCA was used to visualize the relationship between the target and singleton distractor representation only. The formal correlation analysis was performed based on the raw signal space.

### Statistics

We conducted one-sample (one-sided) t-tests to compare decoding accuracies against the theoretical chance level, as below-chance decoding is not meaningful for this analysis. All other comparisons were performed using two-sided t-tests. One-sample t-tests were also used to compare temporal generalizability scores and cross-condition generalizability scores against the theoretical chance level. In both cases, above-chance scores indicated positive generalizability, whereas below-chance scores indicated negative generalizability. Paired t-tests were used to compare prediction probabilities for target locations, singleton distractor locations, and non-singleton distractor locations. Finally, one-sample t-tests were conducted to compare neural correlations between target and singleton distractor representations against zero, with positive values indicating positive correlations and negative values indicating negative correlations. All analyses were performed in Python using standard statistical packages. Bayes factors for the alternative hypothesis (*BF10*) were reported, with values between 1 and 3 indicating anecdotal evidence, values between 3 and 10 indicating moderate evidence, and values greater than 10 indicating strong evidence. To account for multiple comparisons, Sidak correction was applied, and adjusted p-values were reported.

## Significance Statement

A primary goal of attention is to select relevant information and ignore irrelevant information from the external environment. While the mechanism of attentional enhancement has been extensively studied, the mechanism of attentional suppression remains less clear. Here, we show that neural representations of salient singleton distractors undergo a rapid inversion around 200 ms into the search. The inverted distractor representations can be subsequently read out as suppression signals in the computation of spatial priorities. Our findings suggest that the reorganization of initial pop-out distractor signals drives rapid attentional suppression and facilitates goal-directed visual search.
